# Optofluidic vapor sensing with free-space coupled 2D photonic crystal slabs

**DOI:** 10.1038/s41598-019-41048-w

**Published:** 2019-03-12

**Authors:** Yonghao Liu, Shuling Wang, Priyanka Biswas, Prithviraj Palit, Weidong Zhou, Yuze Sun

**Affiliations:** Department of Electrical Engineering, University of Texas at Arlington, Arlington, Texas 76019 USA

## Abstract

We report here a compact vapor sensor based on polymer coated two-dimensional (2D) defect-free photonic crystal slabs (PCS). The sensing mechanism is based on the resonance spectral shift associated with the Fano resonance mode in the PCS due to the vapor molecule adsorption and desorption induced changes in both polymer thickness and polymer refractive index (RI). Sensitivity due to RI and thickness change were theoretically investigated respectively. With three different thicknesses of OV-101 polymer coating, sensitivity and response time were experimentally evaluated for hexane and ethanol vapors. The polymer demonstrated roughly four times higher sensitivity towards the hexane vapor than ethanol vapor. The PCS sensor with thicker polymer coating showed higher sensitivity to both hexane and ethanol vapors but exhibiting longer response time.

## Introduction

Optical vapor sensors have been widely used in industrial emission control, environmental monitoring, healthcare and homeland security^1^. The majority of optical vapor sensors measure either gas absorption or refractive index (RI) change induced by the gases. Absorption-based vapor sensor is highly specific because of the characteristic absorption lines resulting from the molecular rotation and vibration in the near IR (700 nm–2.5 μm) and mid-IR range (2.5–14 μm)^1–9^. However, the sensor typically requires large dimension of the gas cell to have enough absorption based on Beer–Lambert law. This creates significant challenges towards the development of compact on-chip sensors.

The optical RI-based on-chip vapor sensors have been studied extensively for gas detection with different device configurations, including micro-ring resonators^10–13^, surface plasmon resonances (SPR)^14,15^, Fabry-Perot interferometers^12,16–18^, and photonic crystal cavities^19–24^. In these sensors, light interferes constructively at certain wavelength to increase the effective interaction length, which enables compact integration of vapor sensors on chip. Typically, a layer of vapor-sensitive polymer is coated on the sensor surface to capture the vapor molecules and enhances the detection sensitivity^10,11^. However, compared to absorption-based gas sensor, optical RI-based sensor does not produce a unique response to a particular vapor analyte. The lacking of detection specificity is a significant challenge in RI-based vapor sensors, which must be addressed by other technologies, such as gas chromatography (GC)^12,25,26^.

In our previous work, bulk liquid sensing based on free-space coupled Fano resonance defect-free photonic crystal slabs (PCS) has been demonstrated^27–29^. Fano resonance in the defect-free PCS provides an effective channel for the light to radiate to the external environment^30,31^, and the resonant wavelength is highly sensitive to the RI change near the PCS surface. A detection limit (DL) of 10^−5^ to 10^−6^ refractive index units (RIU) has been demonstrated for bulk liquid sensing^27–29^. In bulk liquid sensing, the liquid near the sensor surface is considered as a homogenous material with infinite thickness. However, in PCS vapor sensors, polymer with a finite thickness is used on the PCS surface to facilitate sensing, which is a key difference from our previous work.

In this work, we report the free-space coupled 2D defect-free PCS vapor sensor. The PC sensor described here has a small footprint (*e*.*g*., 500 × 500 μm^2^), which can be conveniently integrated with microfabricated columns in a typical micro-GC system as an on-column gas detector^12,25,26^. The 2D PCS structures are compatible with the complementary metal-oxide-semiconductor (CMOS) fabrication process, making it possible for mass production in large-scale integrated photonics chips. A thin layer of non-polar polymer, dimethylsilicone (OV-101), was coated to the sensor surface to facilitate vapor adsorption and desorption. The sensitivity and response time of the sensor were systematically characterized. Polymer thickness is also investigated to optimize the sensing performance.

## Sensor Design and Fabrication

**Figure 1:**
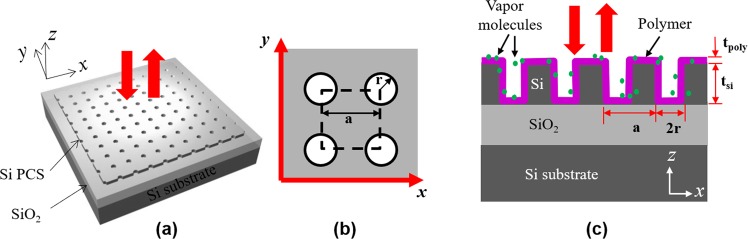
(**a**) Schematic view of a PCS vapor sensor with free-space coupled laser beam. (**b**) Device top view in *x-y* plane. (**c**) Device cross-sectional view in *x-z* plane. The PCS is coated with OV-101 polymer. PCS configuration: *a* is the lattice constant, *r* is the hole radius, *t*_Si_ and *t*_poly_ are the thicknesses of Si and polymer, respectively.

The schematic of the PCS sensor is shown in Fig. 1. The 2D square lattice air hole PCS cavity is fabricated on silicon-on-insulator (SOI) substrate, with light coupled vertically along the surface-normal direction (*z*-axis). A uniform thin layer of polymer is coated on top of the PCS cavity. When vapor molecules are adsorbed by the polymer, the interaction between vapor molecules and polymer causes changes in both RI and thickness of the polymer, resulting in a spectral shift of the Fano resonance.

**Figure 2:**
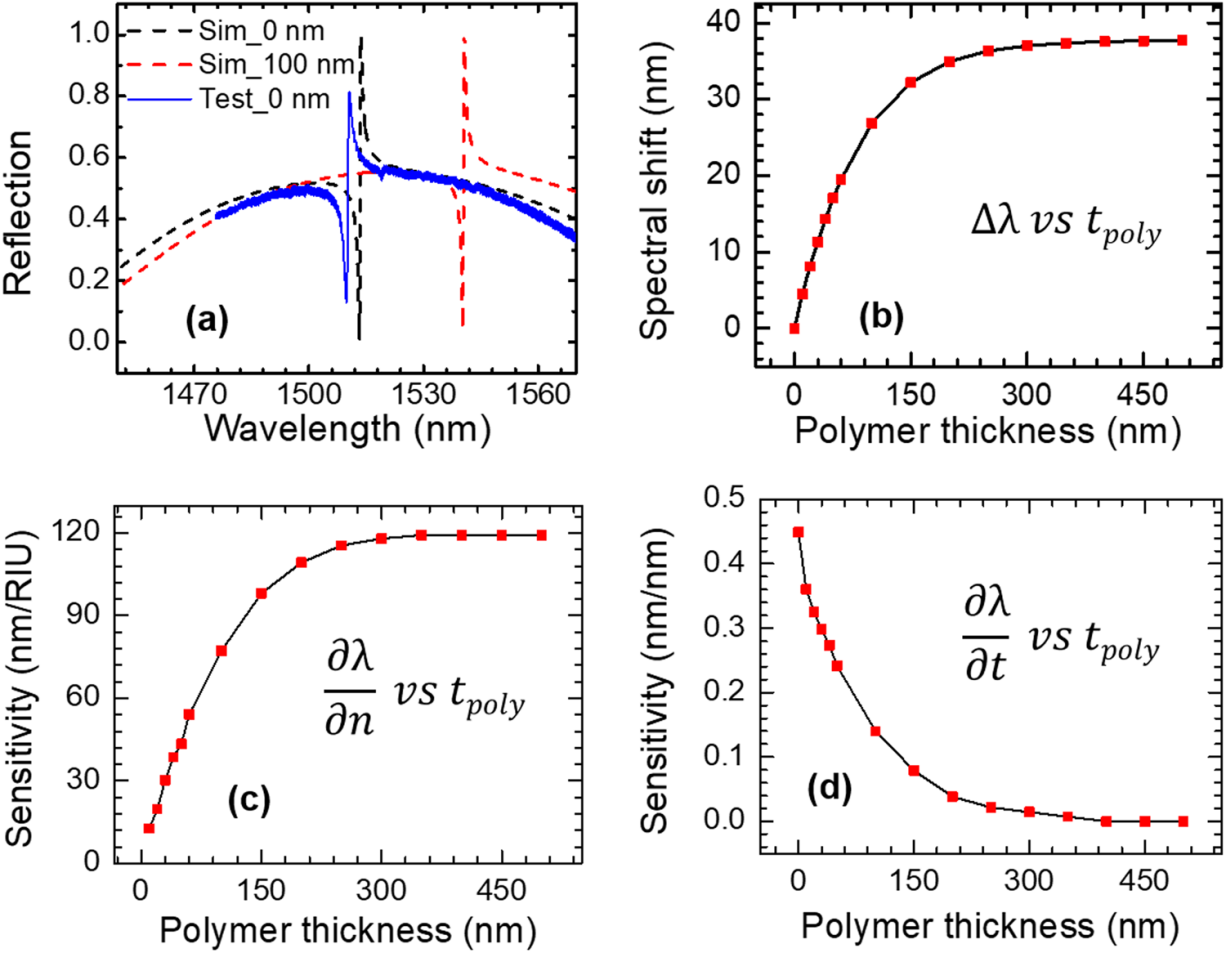
Spectral shift and sensitivity of the PCS in relation to polymer thickness. (**a**) The simulated reflection spectra for PCS without and with 100 nm polymer, and the measured spectrum without polymer. (**b**) Simulated spectral shift of PCS sensor for different polymer thicknesses. (**c**) RI sensitivity ∂*λ*/∂*n* for different polymer thicknesses. (**d**) Thickness sensitivity ∂*λ*/∂*t* for different polymer thicknesses.

The PCS cavity is designed with Stanford Stratified Structure Solver (S^4^) software package^32^. A SOI structure with 250 nm top Si device layer (*t*_Si_) and 3 μm SiO_2_ layer was used in simulation. To tune the center wavelength of a Fano resonance to around 1550 nm, the Si PCS is designed with lattice constant *a* = 980 nm and air hole radius *r* = 85 nm. Different polymer thicknesses (*t*_poly_) were considered for optimal sensing performance. The refractive indices of Si, SiO_2_ and OV-101 polymer are 3.48, 1.45 and 1.4, respectively. The reflection spectra at surface-normal incidence for the PCS without polymer coating (Sim_0 nm) and with 100 nm polymer coating (Sim_100 nm) are shown in Fig. 2(a). The resonant spectral location redshifts 27 nm, from 1513 nm to 1540 nm, due to 100 nm polymer coating on top of the PCS. The spectral shift ∆λ for the PCS with various polymer thicknesses *t*_poly_ is plotted in Fig. 2(b). The resonance shift in relation to polymer thickness ∆λ/∆ *t*_poly_ is 0.33 nm/nm for polymer thickness below 60 nm. The spectral shift saturates at 38 nm when the polymer thickness is greater than 250 nm.

Since the polymer undergoes RI and thickness change when interacting with the vapor molecules, the spectral shift of Fano resonance can be expressed as^11^:

1 $${\rm{\Delta }}\lambda =(\partial \lambda /\partial n)\,\bullet \,{\rm{\Delta }}n+(\partial \lambda /\partial t)\,\bullet \,{\rm{\Delta }}t$$

where *n* and *t* are the RI and thickness of the polymer. ∂*λ*/∂n and ∂*λ*/∂*t* refer to the RI sensitivity (*S*_RI_) and thickness sensitivity (*S*_t_), respectively, which are the intrinsic properties associated with the optical mode of the PCS. The RI sensitivity is calculated by tracking the resonance shift for the PCS structure with a small variation in refractive index of the polymer around 1.4. The RI sensitivity *S*_RI_ (=∂*λ*/∂n) at different polymer thickness is plotted in Fig. 2(c). *S*_RI_ increases linearly in the 0–100 nm polymer range. *S*_RI_ reaches 115 nm/RIU with 250 nm polymer and saturates when thickness increases beyond 250 nm.

The thickness sensitivity *S*_t_ (=∂*λ*/∂n) is plotted in Fig. 2(d). *S*_t_ has high value for small polymer thickness and drops drastically when thickness increases. When the polymer layer is thicker than 250 nm, the thickness sensitivity is close to zero, meaning that the PCS sensor is not sensitive to the change of polymer thickness anymore. In the case of thin polymer coating, the RI sensitivity is much lower than the thickness sensitivity. For thicker polymer, the RI sensitivity dominates.

The field distribution was simulated for the PCS with 50 nm polymer and 300 nm polymer to represent the case of thin polymer coating and thick polymer coating, respectively. Field distribution is computed with finite-difference time-domain (FDTD) software package of MEEP^33^.

**Figure 3:**
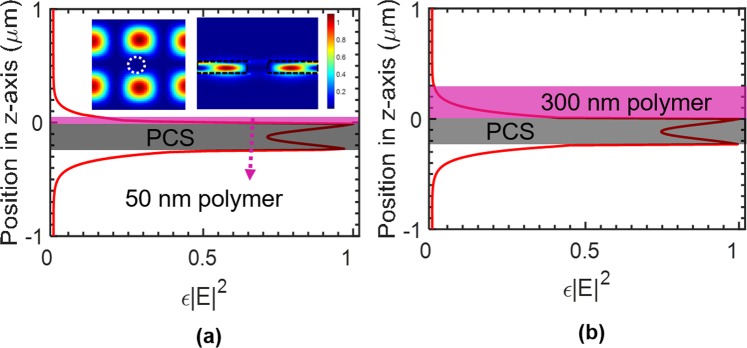
Distribution of integrated *ε*|*E*|^2^ in one unit cell along vertical (*z*-axis) direction for polymer thicknesses of (**a**) 50 nm and (**b**) 300 nm. Shown in the inset of (**a**) are *ε*|*E*|^2^ profiles at the center of the PCS in the *x-y* plane (left) and at the center of the hole in the *x-z* plane (right), with boundary of hole and Si region shown with dashed lines.

The optical overlap integral *f* is defined as the ratio of the electric field energy in the polymer region to the total energy for a given mode^28,34^:

2 $$f=\frac{{\int }_{{V}_{polymer}}\varepsilon {|E|}^{2}dv}{{\int }_{{V}_{total}}\varepsilon {|E|}^{2}dv}$$

where *ε* is the dielectric constant of the material, and *E* is the electric field. The RI sensitivity *S*_RI_ is directly proportional to the optical overlap integral *f* according to *S*_RI_ = ∆*λ*/∆*n* = *f*∙*λ*_0_/*n*, where *λ*_0_ is the resonant wavelength and *n* is the RI of the polymer. To evaluate the amount of field energy in the polymer region, *ε*|*E*|^2^ in one unit cell is integrated along the *z* axis. The results are plotted in Fig. 3(a,b), for 50 nm polymer and 300 nm polymer, respectively. The insets shown in Fig. 3(a) are the *ε*|*E*|^2^ distribution at the center of the Si PCS in the *x-y* plane (left) and at the center of the hole in the *y-z* plane (right). Most of the field energy is concentrated in the high refractive index Si region. The integrated *ε*|*E*|^2^ has the maximum value at the two interfaces of the Si slab, and decays exponentially when moving away from the Si slab interfaces to the polymer layer or buried oxide layer.

The optical overlap integral *f* in the polymer layer are 4.18% and 11.32% for 50 nm and 300 nm thick polymer, respectively. We can derive the *S*_RI_ as 45.3 nm/RIU for 50 nm polymer and 123.4 nm/RIU for 300 nm polymer. The RI sensitivities calculated from field distribution match well with those calculated from spectral resonance tracking, as shown in Fig. 2(c). As we can see from Fig. 3(b), there is almost no field outside the 300 nm polymer region, which explains the invariant RI sensitivity and near-zero thickness sensitivity when thickness is beyond 250 nm shown in Fig. 2(c,d).

**Figure 4:**
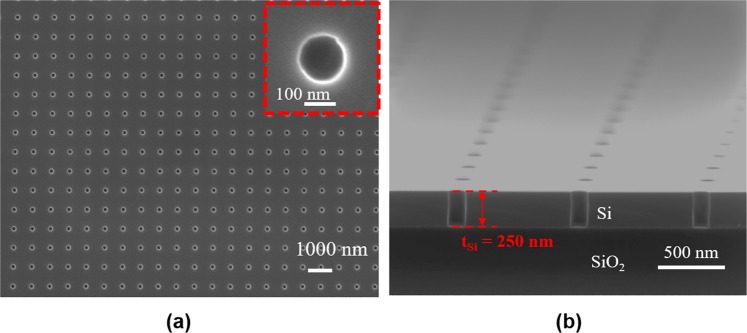
(**a**) Top view and (**b**) Cross-sectional view SEM images of one fabricated PCS sensor on SOI substrate, with *a = *980 nm and *r* = 85 nm.

The top view and cross-sectional view of the scanning electron microscope (SEM) images for one fabricated device are shown in Fig. 4 (a,b), respectively. Three PCS devices with the same design were coated with different thicknesses of OV-101 polymer by controlling the coating solution concentration. The polymer thickness on each device is estimated based on the simulation results shown in Fig. 2(b) by measuring the resonance spectral shift before and after coating. The measured spectral shift indicates a layer of 5 nm (Device #1), 20 nm (Device #2), and 54 nm (Device #3), respectively, was coated on each device. We fabricated the PCS with a footprint of 500 × 500 μm^2^ for easier alignment of the incident beam on the device. The footprint can be further reduced to around 40 × 40 μm^2^ without compromising the quality factor or sensitivity of the PCS.

### Sensor characterization

**Figure 5:**
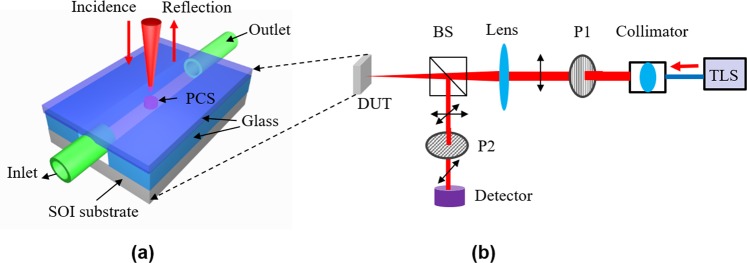
(**a**) A schematic of the PCS vapor sensor device on a chip; (**b**) Reflection measurement setup under surface-normal incidence. TLS: tunable laser source, P1 and P2: linear polarizer, BS: beam splitter, DUT: device under test.

**Figure 6:**
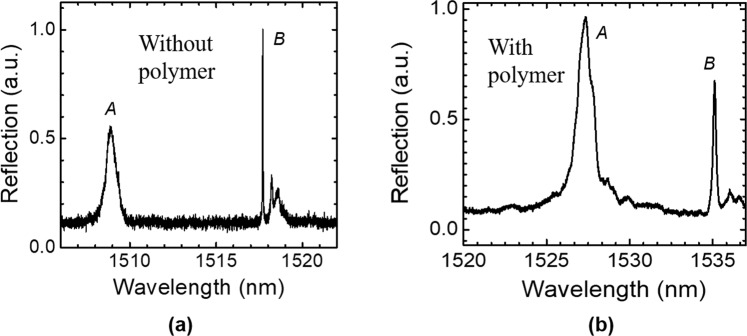
Measured reflection spectrum of Device #3 with cross-polarization technique: (**a**) without polymer coating; and (**b**) with polymer coating.

A glass chamber was built on top of the device for vapor sample delivery, as shown in Fig. 5(a). The inlet and outlet are formed by inserting glass capillaries into the gas cell and sealed with the optical glue. The optical characterization setup is shown in Fig. 5(b). The reflection spectrum (measured without polarizer P2 in Fig. 5(b)) of Device #3 without polymer coating is shown in Fig. 2(a). Cross polarization technique can reveal symmetry-protected bound states in the continuum (BIC) modes, as discussed in our previous work^28,29^. The reflection spectra of Device #3 measured with cross-polarization technique, without and with polymer coating, are shown in Fig. 6(a,b), respectively. Mode *A* corresponds to the resonance mode shown in Fig. 2(a). Mode *B* is the symmetry-protected BIC mode and it can be observed due to the effective small inherent incident angle of the beam^28^. A redshift of 18 nm were observed for both Mode *A* and *B* after polymer coating. The polymer thickness was derived to be 54 nm based on the simulation results shown in Fig. 2(b). The polymer thickness of Device #1 and Device #2 were estimated using the same method.

**Figure 7:**
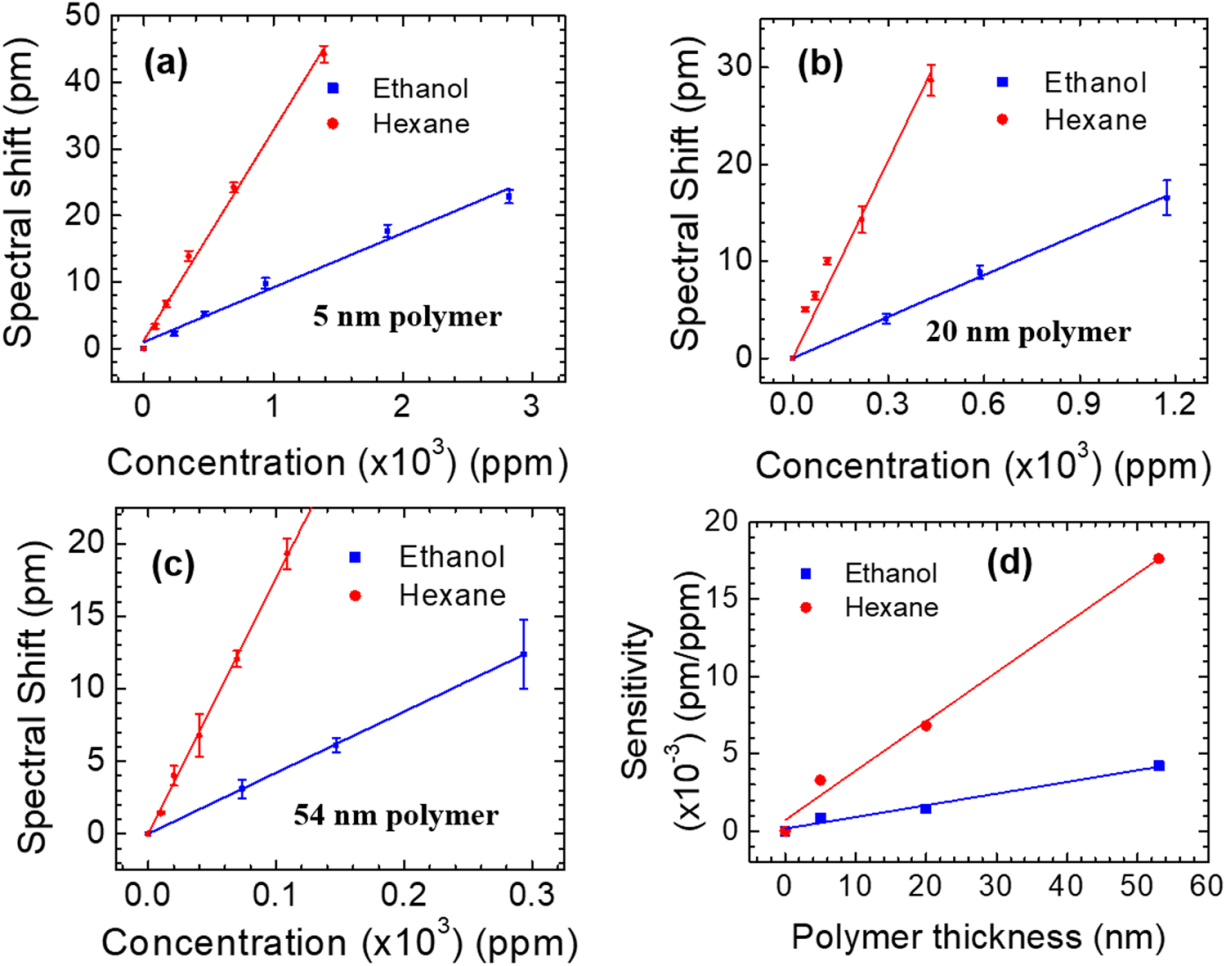
Measured spectral shift of the resonant mode at various hexane and ethanol concentrations for the PCS coated with polymer of three different thicknesses: (**a**) Device #1 with 5 nm polymer; (**b**) Device #2 with 20 nm polymer; and (**c**) Device #3 with 54 nm polymer. (**d**) Sensitivity of the PCS to hexane and ethanol vapor with different polymer coating thicknesses.

To study the chemical vapor sensing, hexane and ethanol vapors are utilized to represent nonpolar and polar analytes respectively. We tested mode *B* because it has a higher quality factor, while the sensitivity of mode *A* and mode *B* are similar at different polymer thicknesses based on our simulation results. Detailed discussion about these modes can be found in a previous work^28^. The spectral shift obtained at equilibrium state for different concentrations of ethanol or hexane vapor for three devices are plotted in Fig. 7(a–c). Device #1 with 5 nm polymer coating exhibits a sensitivity of 3.29 × 10^−3^ pm/ppm for hexane vapor and 8.7 × 10^−4^ pm/ppm for ethanol vapor, as shown with the linear fitted curves in Fig. 7(a). The refractive index of polymer increases linearly when vapor molecule concentration increases, if small index changes are considered^35^. Device #2 with 20 nm polymer coating shows an higher sensitivity of 6.81 × 10^−3^ pm/ppm to hexane vapor and 1.43 × 10^−3^ pm/ppm to ethanol vapor, respectively, as shown in Fig. 7(b). The sensitivity of hexane vapor and ethanol vapor, respectively, for device #3 with 54 nm polymer coating is fitted to be 1.762 × 10^−2^ pm/ppm and 4.22 × 10^−3^ pm/ppm, as shown in Fig. 7(c). Limited by 1 pm spectral resolution of the tunable laser source, the detection limit for hexane vapor is estimated to be 57 ppm for Device #3.

The sensitivity of hexane vapor is approximately four times higher than that of ethanol vapor, which is consistent over all three devices. This is expected since OV-101 is a nonpolar polymer and has higher solubility for nonpolar vapor molecules such as hexane. Shown in Fig. 7(d) is the extracted sensitivity for devices with different polymer coating thicknesses. The sensitivity of hexane and ethanol increase linearly with polymer thickness in the range of 0–50 nm.

**Figure 8:**
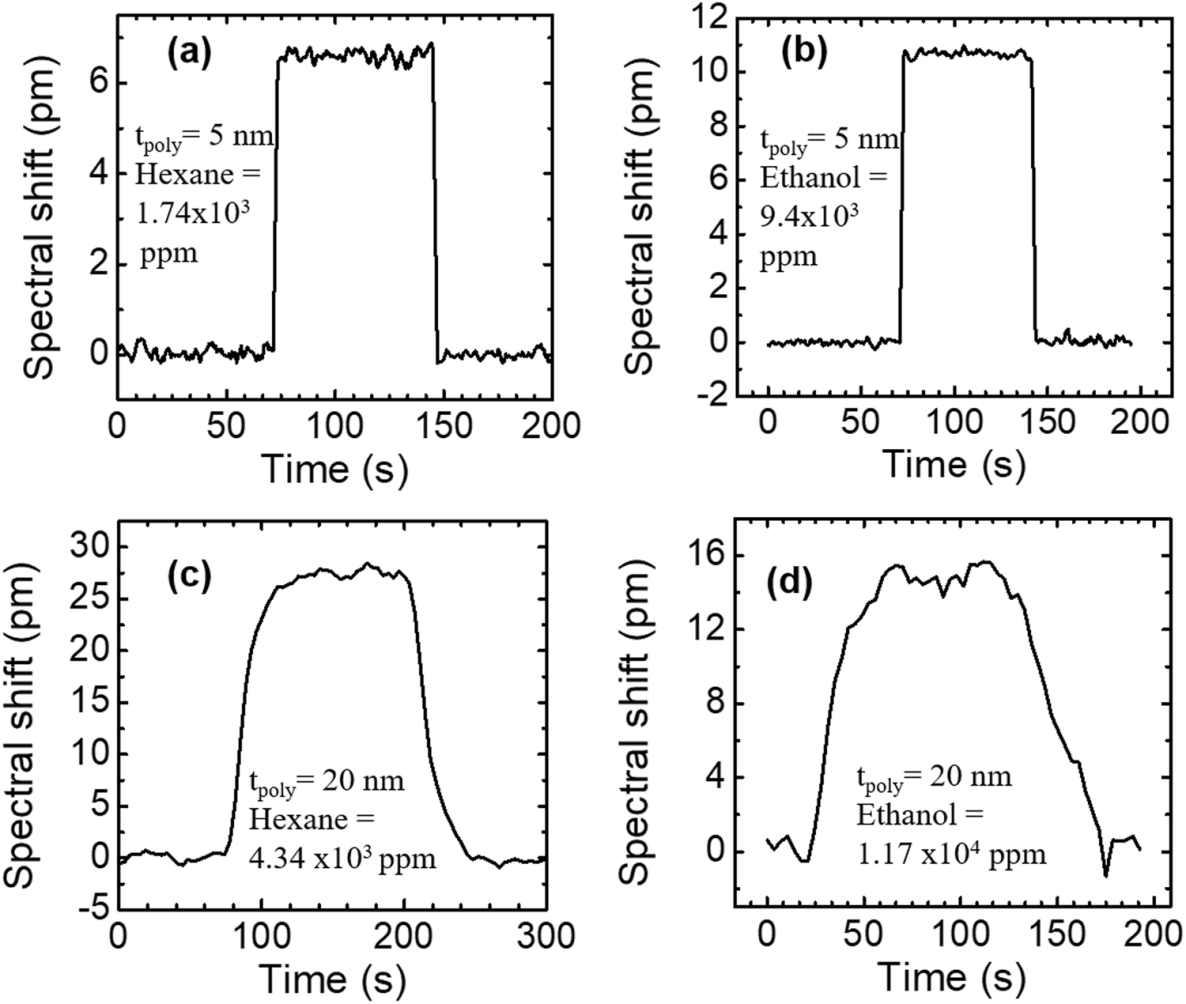
Measured sensorgrams of hexane and ethanol vapors for the PCS coated with two polymer thicknesses: (**a**) 1.74 × 10^3^ ppm hexane vapor with 5 nm polymer; (**b**) 9.4 × 10^3^ ppm ethanol vapor with 5 nm polymer; (**c**) 4.34 × 10^3^ ppm hexane vapor with 20 nm polymer; and (**d**) 1.17 × 10^4^ ppm ethanol vapor with 20 nm polymer.

The measured sensorgrams for Device #1 (with 5 nm polymer coating) in 1.74 × 10^3^ ppm hexane and 9.4 × 10^3^ ppm ethanol are shown in Fig. 8(a,b), respectively. The resonance mode quickly returns to the baseline after purging with air, indicating that the vapor molecules diffused out of the polymer completely and the polymer is fully regenerated. The response time is defined as the amount of time to reach the equilibrium state (maximum spectral shift) at one concentration. The response time to the vapor analyte and sensor regeneration time are within 2 seconds for Device #1 with 5 nm polymer coating. The sensorgrams for Device #2 (with 20 nm polymer coating) in 4.34 × 10^3^ ppm hexane vapor and 1.17 × 10^4^ ppm ethanol vapor are shown in Fig. 8(c,d), respectively. The response time to vapor analyte and the sensor regeneration time are around 50 seconds.

**Figure 9:**
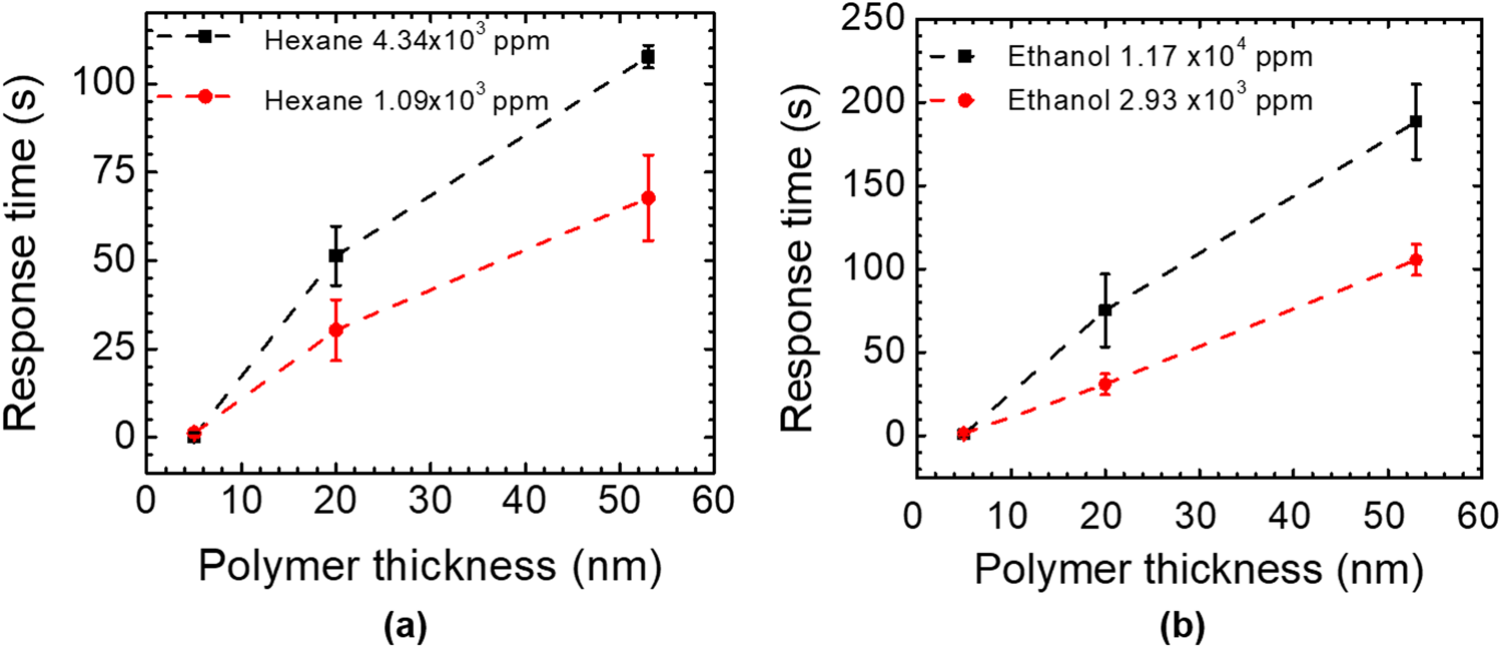
Measured sensor response times with different polymer coating thicknesses: (**a**) Hexane vapor with concentrations of 1.09 × 10^3^ ppm and 4.34 × 10^3^ ppm; and (**b**) Ethanol vapor with concentrations of 2.93 × 10^3^ ppm and 1.17 × 10^4^ ppm.

The response times of the three devices with 5 nm, 20 nm, and 54 nm polymer coating to hexane vapors (1.09 × 10^3^ ppm and 4.34 × 10^3^ ppm) are plotted in Fig. 9(a), while the response time of the three devices to ethanol vapors (2.93 × 10^3^ ppm and 1.17 × 10^4^ ppm) are shown in Fig. 9(b). According to Fick’s laws of diffusion, the response time for thicker polymer is longer, because it takes more time for the vapor molecules to diffuse into a thicker polymer to reach the equilibrium^15^. As shown in Fig. 9(a,b), higher concentration of vapor requires longer time for the sensor to reach the equilibrium state than lower concentration of vapor, because the diffusion coefficient might be concentration dependent at higher concentrations^35^.

## Discussion

Free-space coupled optical vapor sensor on SOI was demonstrated based on compact 2D defect-free Fano resonance PCS cavities. The impact of the polymer coating thickness was investigated theoretically and experimentally. The sensitivity has a linear dependence for the polymer thickness less than 54 nm and reaches saturation for polymer thickness greater than 300 nm, which agrees well with the engineered Fano resonance field distributions at PCS surface. A detection limit of 57 ppm to hexane vapor was observed for the sensor with 54 nm polymer coating, which is mainly limited by the intrinsic spectral resolution of the measurement setup. There exists a trade-off between the sensitivity and the response time. Thicker polymer tends to have higher sensitivity but responds slower to the vapor according to Fick’s laws of diffusion. To avoid the trade-off between the sensitivity and the response time, the design of the PCS structures can be further explored to have a more desirable optical filed distribution profile near the PCS surface, *i*.*e*., larger field distribution in the polymer layer and highly concentrated near the sensor surface. The sensor with non-polar polymer coating showed four times higher sensitivity of the non-polar hexane vapor than that of the polar ethanol vapor. 2D PCS based vapor sensor can be integrated with μGC system to detect and analyze multiple vapors. The sensor can operate with free-space coupled light beam, with more tolerance in the alignment for device operation. The platform could be useful in a variety of industrial process control, environmental monitoring, and biomedical research.

## Methods

The PCS sensor on SOI was fabricated with standard electron beam lithography (EBL) process and reactive ion etching (RIE) process, as detailed in our previous work^27,28,36^. OV-101 (Ohio Valley Specialty) was used as vapor sensing polymer. The polymer coating protocol was adapted based on well-developed coating procedures for gas separation columns^10,37^. In short, the coating solution of certain concentration was flowed into the glass chamber built on top of the PCS and left to incubate for 10 min. The solution was subsequently removed by applying vacuum at the device outlet overnight. Vapor samples were prepared in a 10 L Tedlar bag by injecting a small volume of sample liquid and letting it fully evaporates. The sample was further diluted from the bag using dry air to the desired concentrations. Various concentrations of analyte, from 100 to 3 × 10^4^ ppm (parts per million), were injected into the gas chamber by a syringe pump at a flow rate of 0.5 mL/min. Air was used to purge the vapor analyte out of the glass chamber to re-establish the sensing baseline after each sensing event.

Reflection spectra of the PCS devices were measured by scanning the wavelength of a tunable laser light source (Agilent 81980A). The device was mounted on a translation stage and the beam shines on the device under surface-normal incidence condition. The reflected beam was measured by a germanium detector (Agilent 81623B), with a noise level of 100 pW. In order to suppress the Fabry-Perot interference induced by the cover glass, cross-polarization technique was used^28,29^. The measurement setup was controlled with LabVIEW program. Each reflection spectrum was fitted with Lorentzian function to obtain the center wavelength of the resonance. The temperature was 21.6 degree during experiment, with variation around 0.1 degree. The effect of small variation in temperature was minimized by doing a baseline correction in resonance tracking.

## Data Availability

Data presented in this study is available from the corresponding authors upon reasonable request.
